# Green Hospital Awareness: Evidence from Healthcare Technician Students

**DOI:** 10.3390/healthcare14060723

**Published:** 2026-03-12

**Authors:** Ayşegül Doğan Kaya, Arzum Çelik Bekleviç

**Affiliations:** 1 Department of Dental Services, Ahmet Erdoğan Vocational School of Health Services, Zonguldak Bülent Ecevit University, Zonguldak 67000, Turkey; 2 Department of Medical Services and Techniques, Ahmet Erdoğan Vocational School of Health Services, Zonguldak Bülent Ecevit University, Zonguldak 67000, Turkey; arzum.cb@beun.edu.tr

**Keywords:** green hospital, sustainability awareness, sustainable health systems

## Abstract

**Background/Objectives**: Climate change and environmental degradation pose increasing public health threats, while healthcare systems significantly contribute through high energy use, water consumption, and waste generation. In hospital settings, healthcare technicians play a critical role, as their routine practices directly influence environmental sustainability. Despite this central role, healthcare technicians remain an under-recognized group in sustainability research and health policy. Green hospital practices therefore constitute a key public health strategy, requiring strategic management attention to this operational workforce. This study aimed to assess green hospital awareness among healthcare technician students in Türkiye and to examine associated sociodemographic factors from a public health perspective. **Methods**: This descriptive cross-sectional study included 313 students enrolled in health services vocational programs who were receiving hospital-based practical training. Data were collected using a personal information form and the validated Green Hospital Awareness Scale. Descriptive statistics, independent *t*-tests, one-way ANOVA, and Spearman correlation analyses were conducted using SPSS version 26.0, with statistical significance set at *p* < 0.05. **Results**: Although most participants reported no prior knowledge of the green hospital concept, overall awareness levels were moderate to high. The highest mean scores were observed in indoor environmental quality and materials–resources subdimensions, whereas water efficiency scores were the lowest. Female students demonstrated significantly higher total awareness and materials–resources scores (*p* < 0.05). No significant differences were observed by age or academic department. **Conclusions**: Healthcare technician students exhibit measurable green hospital awareness; however, important gaps remain in resource- and infrastructure-related sustainability domains. Strengthening educational and policy initiatives targeting this often-overlooked yet operationally essential workforce may improve environmentally responsible practices, enhance resource efficiency, and support sustainable healthcare systems and population health.

## 1. Introduction

The process of globalization has generated multidimensional impacts on both human life and the global ecosystem, producing positive and negative outcomes simultaneously. Climate change resulting from global warming is recognized as a major challenge of the current century and is considered a key driver of natural disasters and a wide range of diseases [1,2]. Imbalances between carbon emissions and absorption, increasing air and environmental pollution, waste generation, and the prolonged use of energy resources are among the primary factors contributing to global warming [3]. Owing to the nature of the services they provide, healthcare institutions are among the sectors with the highest levels of carbon emissions, energy consumption, and waste generation [4]. In this context, healthcare facilities, which are characterized by intensive energy use, are directly linked to the environmental impacts of globalization. Greenhouse gas emissions and the carbon footprint associated with the healthcare sector begin with hospital construction and continue to increase throughout service delivery, ultimately becoming a significant public health concern due to the extensive consumption of natural resources [5]. Indeed, Health Care Without Harm emphasizes the scale of emissions generated by the healthcare sector by stating that, if the global healthcare sector were considered a country, it would rank as the fifth-largest contributor to greenhouse gas emissions and carbon footprint worldwide. Furthermore, in its 2019 report, the organization reported that greenhouse gas and carbon emissions originating solely from healthcare institutions account for approximately 4.4% of global climate impacts, corresponding to an estimated 2 gigatons of CO_2_ emissions [6,7].

It is highly ironic that institutions whose primary mission is to protect and maintain health simultaneously contribute to environmental degradation that may ultimately threaten health in the future [4,8]. Moreover, given that healthcare facilities operate on a 24/7 basis, continuous energy-intensive activities—such as lighting, ventilation, and the operation of medical equipment—along with waste generated during patient care and the release of anesthetic gases, consistently contribute to escalating environmental hazards [2,4].

All these factors have given rise to the concept of “green buildings.” The green building approach, which has gained widespread adoption globally, has prompted hospitals to reconsider traditional design and operational models and has led to the increasing adoption of green hospital practices [9]. This trend is driven by several factors, including the intensive use of energy and water, substantial waste generation, high material demands, and, in many cases, inadequate indoor environmental conditions within hospital settings. In this context, healthcare facilities that aim to reduce natural resource consumption, limit waste generation, and provide healthier and higher-quality physical environments for patients are defined in the literature as “green hospitals” [10,11]. Although primarily focused on environmental protection, this concept emphasizes sustainability in building design and the reduction of carbon footprint during healthcare service delivery [3,12,13]. From a broader perspective, the literature highlights the critical role of healthcare workers in advocating environmentally friendly behaviors, promoting sustainability efforts to policymakers and the public, encouraging sustainable practices within healthcare services, and adopting behaviors and attitudes aimed at reducing the carbon footprint [14,15].

The establishment of structured, context-specific checklists that define standards for waste disposal, water efficiency, energy conservation, reduction of environmental harm, and recycling practices [16], together with healthcare workers awareness and internalization of green hospital principles, plays a direct role in advancing environmental sustainability in healthcare settings [17,18]. Within this framework, healthcare workers emerge as essential agents of decarbonization efforts in the health sector [19]. Notably, healthcare technicians occupy a particularly critical position in translating sustainability strategies into daily practice. Their routine responsibilities—such as the segregation of medical waste at the source, safe storage and transportation of hazardous materials, prevention of environmental contamination, reduction of infection risks, and the handling and use of medical supplies—directly shape hospitals’ environmental performance. As frontline practitioners who operate at the intersection of clinical care and hospital operations, healthcare technicians constitute one of the most influential yet often underrecognized professional groups in sustainable healthcare delivery, following physicians and nurses. In Türkiye, recent data indicating a workforce of approximately 290,415 healthcare technicians highlight the substantial scale and strategic importance of this group within the healthcare system [20]. A review of the existing literature indicates that studies focusing on green hospital awareness remain limited in number. In Türkiye, research in this field has predominantly concentrated on hospital managers [21] as well as patients and nurses [22,23,24]. However, considering that healthcare technicians’ awareness of green hospital practices can contribute positively to the institutions in which they work, fostering this awareness during the educational period is of critical importance. In this context, the present study was conducted to determine the level of awareness regarding green hospital practices among vocational school students in health services who participate in hospital-based practical training, as well as to identify the sociodemographic factors influencing this awareness.

## 2. Materials and Methods

### 2.1. Study Design

This study was conducted as a descriptive cross-sectional investigation. The study population comprised students enrolled in health services vocational programs who were undertaking hospital-based practical training. The total number of students participating in hospital practice across the relevant departments was 525. Using a 95% confidence level, a 5% margin of error, and an assumed proportion of *p* = 0.5, the required sample size was calculated as 223 based on the finite population formula. Ultimately, the study was completed with 313 students who voluntarily consented to participate.

### 2.2. Data Collection

Data were collected from students enrolled in the Health Services Vocational School. Participation was voluntary, and eligibility criteria included being currently enrolled in the program and actively engaged in hospital-based practical clinical training at affiliated hospitals. Data collection was conducted exclusively via an online questionnaire developed using Google Forms to ensure accessibility and standardization. Before the questionnaires were administered, participants were informed about the study through face-to-face meetings, and their informed consent was obtained. The data collection period extended over three months, from October to December 2025. Students who were not enrolled in the program at the time of data collection, were not participating in practical training, or declined to participate were excluded from the study.

### 2.3. Data Collection Tools

Data were collected using a personal information form and the House of Quality Green Design (HOQGD) Scale [25]. The scale was originally developed by Wood, Wang, Abdul-Rahman, and Abdul-Nasir (2016) [25] and subsequently adapted into Turkish with demonstrated validity and reliability by Mansur and Korkmaz (2020) [26] under the title Green Hospital Design Scale. The instrument comprises two sections and five subdimensions (Energy efficiency, indoor environmental quality, sustainable site planning, materials and resources, water efficiency), with an overall reliability coefficient (Cronbach’s alpha) of 0.940 reported in the original validation study [26].

The first section includes items designed to assess healthcare users’ awareness of green hospital practices. Participants’ level of agreement with each statement is rated on a five-point Likert scale ranging from 1 (“Never”) to 5 (“Always”). The scale comprises a total of 24 items grouped under five subdimensions. In the present study, internal consistency was evaluated using Cronbach’s alpha coefficients. The overall Cronbach’s alpha value for the scale was found to be 0.949. The alpha coefficients for the subdimensions ranged from 0.749 to 0.936. These findings indicate a high level of reliability for both the overall scale and its subdimensions.

Data were collected on a voluntary basis using a face-to-face questionnaire method. Prior to data collection, participants were informed about the purpose of the study, and informed consent was obtained from all participants.

### 2.4. Statistical Analysis

Construct validity of the scale was examined through Confirmatory Factor Analysis (CFA). The analysis was conducted using IBM SPSS AMOS 24 software and the measurement model was specified in accordance with the theoretical structure of the scale, consisting of five factors and 24 items. In the specified model, each item was allowed to load only on its corresponding latent factor, while the correlations among the latent factors were freely estimated.

Data analysis was performed using IBM SPSS version 26.0. Descriptive statistics were presented as frequencies, percentages, means, and standard deviations. The distributional properties of the total scale score and subdimension scores were assessed by examining skewness and kurtosis values. The skewness and kurtosis values for both the total scale score and its subdimensions ranged between −1.5 and +1.5, indicating that the data followed a normal distribution. Although the age variable did not exhibit a normal distribution, this did not affect the validity of the analyses, as age was not used as a dependent variable in the study.

In the statistical analyses; an independent samples *t*-test was used to compare total scale and subdimension scores according to gender and prior exposure to green hospital education (two-group variables). One-way analysis of variance (One-Way ANOVA) was applied to examine differences based on academic department (more than two groups), and the homogeneity of variances was assessed using Levene’s test. As the age variable did not follow a normal distribution, the relationships between age and the total scale score as well as subdimension scores were analyzed using Spearman’s rank correlation coefficient. Statistical significance was set at *p* < 0.05 for all analyses.

## 3. Results

### 3.1. Descriptive Statistics

A total of 313 graduating students participated in the study. The mean age of the participants was 19.93 ± 3.86 years, and the majority of the sample consisted of female students (87.2%). Most participants were enrolled in the Medical Services and Techniques program (40.6%). Notably, 68.4% of the students reported that they had not previously heard of the concepts of “green hospital” or “green healthcare services” (Table 1).

### 3.2. Confirmatory Factor Analysis

A confirmatory factor analysis (CFA) was conducted to test the five-factor structure of the 24-item scale. The results of the analysis showed that the standardized factor loadings ranged from 0.40 to 0.90, indicating acceptable levels. In addition, the correlation coefficients among the factors ranged from 0.65 to 0.96, suggesting positive relationships between the factors.

The results indicated that the model demonstrated acceptable goodness-of-fit indices (χ^2^/df = 2.610; GFI = 0.855; IFI = 0.924; TLI = 0.911; CFI = 0.923; RMSEA = 0.072). Overall, these findings suggest that the five-factor model was adequately supported and that the measurement model exhibited an acceptable level of construct validity. These findings confirm the five-factor structure of the scale and demonstrate that its construct validity is supported (Table 2).

### 3.3. Validity and Reliability of the Scale

The internal consistency of the scale was high, with a Cronbach’s alpha coefficient of 0.949. The alpha values for the subdimensions ranged from 0.749 to 0.936. These findings indicate that both the overall scale and its subdimensions demonstrate a high level of reliability (Table 3). The very high Cronbach’s alpha values observed for both the subdimensions and the overall scale indicate strong reliability. However, such high values may suggest that some items contain highly similar content, which should be considered in terms of the distinctiveness of the subdimensions and the conceptual diversity they are intended to measure. In this study, factor scores and the Turkish validity were established based on the existing items, so no modifications were made; nonetheless, the elevated Cronbach’s alpha values represent a noteworthy finding.

### 3.4. Scores of the Scale Subdimensions

The maximum possible score on the scale was 120 and the participants’ mean total scale score was 79.68 ± 18.78, indicating a high level of green hospital awareness. Analysis of the subdimensions showed that the highest mean scores were observed for indoor environmental quality (22.30 ± 4.86) and materials and resources (18.63 ± 6.19), whereas the water efficiency subdimension (8.73 ± 3.24) demonstrated a comparatively lower mean score (Table 4).

### 3.5. Associations Between Scale Scores and Sociodemographic Variables

Although the age variable did not follow a normal distribution, Spearman correlation analysis revealed no statistically significant associations between age and the total Green Hospital Awareness Scale score or any of its subdimension scores (*p* > 0.05). These findings indicate that green hospital awareness is independent of age in the study population. Consistent with these results, no significant correlations were observed between age and the overall scale score or subdimension scores based on Spearman’s rank correlation analysis. (*p* > 0.05). No statistically significant correlations were found between age and the energy efficiency (r = 0.067, *p* = 0.238), indoor environmental quality (r = −0.006, *p* = 0.910), sustainable site planning and management (r = −0.026, *p* = 0.647), materials and resources (r = −0.013, *p* = 0.823), and water efficiency (r = −0.013, *p* = 0.820) subdimensions, nor with the total scale score (r = −0.006, *p* = 0.918) (Table 5).

An independent samples *t*-test was conducted to determine whether green hospital awareness among health students differed by gender. The results indicated a statistically significant difference in the overall scale score and the water efficiency subdimension (*p* < 0.05). Female students had higher mean scores in both water efficiency and the total scale compared with male students. However, the effect size was very small for water efficiency (d = 0.10) and small for the total score (d = 0.30). No statistically significant differences were found between female and male students in the other subdimensions of the scale, including energy efficiency, indoor environmental quality, sustainable site planning and management, and materials and resources (*p* > 0.05). These findings suggest that green hospital awareness among health students generally does not vary substantially by gender, with only limited differences observed in certain dimensions.

One-way analysis of variance conducted across academic departments revealed no statistically significant differences in either the total scale score or any of the subdimension scores (*p* > 0.05). Nevertheless, the effect size estimates based on Eta Squared (η^2^) showed very small effects for energy (η^2^ = 0.012), indoor environment (η^2^ = 0.001), sustainable site (η^2^ = 0.001), water efficiency (η^2^ = 0.008), and the total scale score (η^2^ = 0.003), while a small effect was observed for the material resources sub-dimension (η^2^ = 0.021). This finding indicates that green hospital awareness levels do not vary according to the department of study. Furthermore, students who reported prior familiarity with the concepts of green hospitals or green healthcare services demonstrated significantly higher scores in the energy efficiency and materials and resources subdimensions compared to those without such prior knowledge (*p* < 0.05). The effect sizes remained small (d = 0.18–0.25). Overall, although some statistically significant differences were observed, the magnitude of these effects was generally small or very small. No statistically significant differences were observed between the groups with respect to the remaining subdimensions or the total scale score (Table 6).

The results indicate that students’ awareness of green hospitals is primarily shaped through visual, physical, and directly experiential applications; however, awareness regarding applications requiring infrastructure, engineering, and environmental sustainability remains relatively low.

## 4. Discussion

According to the findings of this study, 68.4% of the students reported that they had never heard of the concept of green hospitals; however, the overall mean score obtained from the scale was relatively high. This apparent discrepancy suggests that many students may already engage in environmentally responsible behaviors without explicitly recognizing these behaviors as components of sustainability or green hospital practices. One possible explanation is that the scale items primarily assess routine professional behaviors, such as resource conservation and waste management, which students may perceive as integral elements of good clinical practice rather than as indicators of green hospital awareness. In addition, implicit learning during clinical training may play a role. Students may adopt environmentally responsible practices through institutional routines and role modeling by healthcare professionals, even in the absence of formal instruction or conceptual labeling as “green practices.” This gap between conceptual knowledge and behavioral performance suggests that while students demonstrate positive practices, explicit sustainability education is still needed to enhance awareness, critical understanding, and intentional application of green hospital principles in healthcare settings. Examination of the sub-dimensions of the scale revealed that students obtained the highest mean scores in the sub-dimensions of indoor environmental quality and materials and resources, whereas the water efficiency sub-dimension had a relatively lower mean score. Similarly, Hoşgör and Güngördü [27] found that 70% of university students enrolled in the medical documentation and secretarial program were unaware of the green hospital concept. In the same study, evaluation of the scores obtained from the sub-dimensions of the scale indicated that students demonstrated high awareness regarding energy efficiency, indoor environmental quality, materials and resources, water efficiency, and sustainable site planning. In contrast, a study conducted in India with 180 postgraduate students, focusing on strategies to reduce the carbon footprint, reported that more than half of the participants had a low level of awareness about their carbon footprint [28].

In the international literature, studies on awareness of green hospitals and green healthcare services have predominantly focused on nurses. Various studies examining nurses’ awareness of environmental sustainability have indicated that nurses possess a moderate level of awareness regarding sustainability practices in hospitals and the health impacts of climate change [29,30]. Similarly, a study conducted in Taiwan observed that although nursing students exhibited positive attitudes toward sustainability, their knowledge and behaviors were found to be insufficient [31].

The healthcare sector is among the largest consumers of water outside the commercial sector. Achieving water conservation in hospitals requires reducing unnecessary usage, preventing water wastage, controlling the disposal and reuse of wastewater, and monitoring the water cycle, all of which constitute the essential requirements of sustainable water management [32].

In this study, the low scores of healthcare technician students in the water efficiency sub-dimension of the scale are considered an important finding. As this professional group is responsible for technical tasks and the overall clinical operations in hospitals, they play a significant role in water use, making their awareness of this issue particularly important. This suggests that students tend to develop awareness primarily of green practices that can be observed and experienced in daily practice, whereas their awareness of infrastructure, engineering, and system-based applications remains limited. The present study revealed that student awareness was lowest for infrastructure-dependent practices, such as water efficiency measures and rainwater harvesting. The lower awareness scores observed in the water efficiency dimension can be attributed to several factors. First, the invisibility of water infrastructure within hospital settings limits students’ direct observation and engagement with water-related practices. Second, water efficiency topics are often underrepresented in the educational curriculum, reducing students’ exposure to the principles and importance of water conservation. Additionally, opportunities for direct exposure to such practices during clinical training are often limited, making it difficult for students to observe or engage with these technical aspects of sustainable healthcare operations.

The lower levels of awareness observed in technical areas such as water efficiency are consistent with findings from other studies in healthcare settings. For example, research in hospital and clinical environments has shown that staff often exhibit limited knowledge of water-saving technologies and practices, despite general environmental awareness (Kameli et al., 2025) [33]. These studies suggest that technical or infrastructure-dependent sustainability practices tend to receive less attention in both formal education and workplace training, which may help explain the patterns observed in the present study.

To enhance the practical relevance of the present findings, more specific curricular and workplace-based interventions are recommended. At the curricular level, sustainability-related courses may be strengthened by incorporating dedicated modules on water efficiency, including topics such as water footprint assessment, water consumption monitoring, and efficiency-oriented technologies. These modules could be delivered through applied instructional approaches, such as case-based learning, simulations, and project-based assignments, to support the development of practical competencies.

At the workplace level, in-service training programs focusing on water efficiency appear particularly warranted. Such programs may include hands-on training on water-saving technologies, routine monitoring of water use, leak detection, and preventive maintenance practices. In addition, continuous training initiatives aimed at fostering pro-environmental behaviors among employees may help translate sustainability knowledge into sustained organizational practices. The effectiveness of these educational interventions can be evaluated using a combination of formative and summative assessment strategies. At the curricular level, pre- and post-intervention measures may be used to assess changes in learners’ knowledge, attitudes, and perceived competencies related to water efficiency. Performance-based assessments, such as case analyses, simulations, or applied projects, may further capture the extent to which sustainability knowledge is translated into practice. At the workplace level, training effectiveness could be assessed through objective indicators, including reductions in overall water consumption, improvements in water-use efficiency and adherence to water-saving protocols. Finally, the effectiveness of these educational interventions should be systematically evaluated, using both formative and summative assessment methods, to ensure that students not only acquire knowledge but are also able to apply sustainable practices in clinical settings

The absence of a significant relationship between age and green hospital awareness indicates that awareness levels do not vary according to age; however, a significant difference was observed according to gender. Female students demonstrated significantly higher overall awareness, particularly in the “Materials and Resources” subdimension, a finding that is consistent with previous research indicating that women tend to exhibit stronger pro-environmental attitudes and behaviors across various contexts, including healthcare settings. Specifically, Lee et al. (2013) reported that women were more inclined to engage in energy-saving practices, supporting the notion that gender differences in sustainability behaviors may be consistent across different environmental domains [34]. Beyond general environmental sensitivity, sociocultural and educational factors may also contribute to this difference, such as gendered expectations around care and responsibility or variations in exposure to sustainability content. Additionally, educational experiences or prior exposure to environmental topics could differ by gender, shaping students’ awareness and engagement with sustainable practices When interpreting the findings of the present study, certain methodological considerations should be taken into account. The sample was characterized by a pronounced gender imbalance, with female participants constituting 87.2% of the total sample. Although this distribution reflects the demographic composition of the relevant academic programs in Turkey, the relatively small number of male participants (n = 40) limits the statistical power and generalizability of the gender-based comparisons. Therefore, the findings related to gender differences, should be interpreted with caution. Future research should explore these potential explanations, ideally using balanced samples and qualitative methods such as interviews or focus groups, to better understand the underlying mechanisms driving gender-related differences in sustainability awareness among healthcare technicians.

In contrast to some previous studies, which found no significant gender differences in carbon footprint awareness but observed variations across students from different academic programs [28] the present study did not identify significant differences in total scale scores or sub-dimensions based on field of study. This suggests that awareness of green hospitals is relatively uniform across disciplines and may reflect the limited integration of environmental sustainability topics in existing curricula in a comprehensive and interdisciplinary manner.

Furthermore, students who were previously familiar with the concepts of green hospitals or green healthcare services demonstrated higher awareness in the energy efficiency and materials and resources sub-dimensions. This finding underscores the positive impact of education and informational interventions on fostering awareness. For many healthcare professionals, the primary barrier to environmental responsibility in the workplace remains a lack of awareness and the capacity to translate knowledge into actionable practices [33,35].

The literature often frames the promotion of sustainability in hospitals as a responsibility primarily assigned to administrators [29,36]. However, as demonstrated in the present study, fostering green awareness among healthcare students before they enter professional practice can encourage the adoption of environmentally responsible approaches in their future careers, thereby contributing to the wider implementation of sustainable practices. Instilling this awareness early is considered a significant achievement, as it has the potential to enhance the efficient use of resources, reduce environmental impacts, and strengthen institutional sustainability in the long term. Future research may also benefit from the use of qualitative research methods to gain a deeper understanding of the patterns identified in this study. In particular, interviews or focus group discussions could be employed to explore the underlying reasons for the lower levels of awareness observed in more technical domains, such as water efficiency. Such qualitative approaches may provide contextual insights into educational experiences, organizational practices, and perceived barriers, thereby complementing the quantitative findings and informing more targeted interventions.

In conclusion, the questionnaire demonstrated very high internal consistency (Cronbach’s α = 0.949), indicating strong reliability. Although this supports the robustness of the instrument, such a high alpha value may also suggest potential item overlap, with some items assessing closely related aspects of the construct. This consideration is important when interpreting the findings and future studies may benefit from further examining the scale structure to reduce redundancy while maintaining comprehensive construct coverage.

### Limitations of the Study

This study has several limitations. First, it was conducted at a single university’s Vocational School of Health Services, which may limit the generalizability of the findings. Second, participants’ knowledge and awareness of green hospital practices were self-reported, introducing the possibility of socially desirable responses. Participants may have reported behaviors that reflect how they perceive themselves as environmentally responsible rather than their actual practices.

Importantly, the study measured awareness rather than actual behavior or competence in clinical settings. High awareness scores do not necessarily indicate equivalent levels of practical application. Future research using observational methods, performance assessments, or practical evaluations is recommended to better understand how sustainability awareness translates into real-world behaviors in healthcare contexts.

## 5. Conclusions

In conclusion, the findings of this study indicate that healthcare students generally exhibit a moderate-to-high level of awareness regarding green hospitals. The high overall mean score on the scale suggests that students possess a certain degree of consciousness concerning sustainable healthcare services and environmentally friendly hospital practices. Analysis of the sub-dimensions revealed that students scored highest in indoor environmental quality and materials and resources, whereas awareness regarding water efficiency was comparatively lower.

To enhance healthcare students’ awareness of green hospital practices, it is essential to integrate sustainability and environmentally friendly healthcare topics into curricula in a more systematic and practice-oriented manner. Strengthening education on energy and water efficiency, as well as infrastructure-based green practices, is expected to support future healthcare professionals in contributing effectively to sustainable healthcare systems. Moreover, studies investigating green hospital awareness among healthcare professionals in Turkey remain limited. Therefore, further research in this area is strongly recommended to better inform policy and educational strategies.

## Figures and Tables

**Table 1:** Distribution of participants’ sociodemographic characteristics.

$\mathrm{Age}\;(\bar{X}\;\pm$ SS)	19.93 ± 3.861	
	*N*	%
Gender		
Female	273	87.2
Male	40	12.8
Department		
Medical Services and Techniques	127	40.6
Dental Services	60	19.2
Healthcare Services	53	16.9
Pharmacy Services	73	23.3
Have you previously heard of the concepts of “green		
hospital” or “green healthcare services”?		
Yes	99	31.6
No	214	68.4

**Table 2:** Goodness-of-Fit Indices for the Five-Factor Model of the HOQGD scale.

	Fit Index Values	Good Fit Values (Acceptable Fit)
χ^2^/sd	2.610	≤3 (4–5)
GFI	0.855	≥0.90 (0.89–0.85)
IFI	0.924	≥0.95 (0.94–0.90)
TLI	0.911	≥0.95 (0.94–0.90)
CFI	0.923	≥0.95 (0.94–0.90)
RMSEA	0.072	≤0.05 (0.06–0.08)

χ^2^/sd = Chi-square/degrees of freedom ratio; GFI = Goodness of Fit Index; IFI = Incremental Fit Index; TLI = Tucker–Lewis Index; CFI = Comparative Fit Index; RMSEA = Root Mean Square Error of Approximation.

**Table 3:** Cronbach’s alpha values for the total scale score and subdimensions.

Scale/Subdimension	Number of Items (n)	Cronbach’s α
Energy efficiency	4	0.749
Indoor environmental quality	6	0.831
Sustainable site planning	5	0.853
Materials and resources	6	0.936
Water efficiency	3	0.827
Total scale	24	0.949

**Table 4:** Total scores for the scale and its subdimensions.

Scale/Subdimension	$\bar{X}$ ± SS	Min–Max
Energy efficiency	12.44 ± 3.34	4–20
Indoor environmental quality	22.30 ± 4.86	6–30
Sustainable site planning	17.57 ± 4.53	5–25
Materials and resources	18.63 ± 6.19	6–30
Water efficiency	8.73 ± 3.24	3–15
Total scale	79.68 ± 18.78	24–120

$\bar{X}$ ± SS = Mean ± Standard Deviation; Min–Max = Minimum–Maximum values.

**Table 5:** Association between age and the Green Hospital Awareness Scale (Spearman correlation).

Scale/Subdimension	r	*p*
Energy efficiency	0.067	0.238
Indoor environmental quality	−0.006	0.910
Sustainable site planning and management	−0.026	0.647
Materials and resources	−0.013	0.823
Water efficiency	−0.013	0.820
Total scale score	−0.006	0.918

r = Pearson correlation coefficient; *p* = *p*-value (statistical significance level).

**Table 6:** Comparison of total and subdimension scale scores according to sociodemographic characteristics.

	Energy Efficiency $\bar{X}$ ± SS	Indoor Environmental Quality $\bar{X}$ ± SS	Sustainable Site Planning & Management $\bar{X}$ ± SS	Materials & Resources $\bar{X}$ ± SS	Water Efficiency $\bar{X}$ ± SS	Total $\bar{X}$ ± SS
Female (n = 273)	12.56 ± 3.26	22.48 ± 4.76	17.73 ± 4.43	18.95 ± 6.04	8.77 ± 3.23	80.48 ± 18.56
Male (n = 40)	11.63 ± 3.79	21.13 ± 5.44	16.48 ± 5.09	16.50 ± 6.85	8.45 ± 3.30	74.18 ± 19.58
*t*	1.66	1.65	1.64	2.35	0.58	1.99
*p*	0.097	0.101	0.102	0.019	0.561	0.047
Cohen’s d	0.29	0.28	0.27	0.10	0.41	0.30
Medical Services and Techniques (n = 127)	12.81 ± 3.35	22.16 ± 4.96	17.55 ± 4.13	19.15 ± 5.89	8.97 ± 3.39	80.64 ± 18.08
Dental Services (n = 60)	12.48 ± 3.33	22.53 ± 5.19	17.33 ± 5.39	17.73 ± 6.83	7.85 ± 3.23	77.93 ± 20.55
Healthcare Services (n = 53)	11.81 ± 3.54	22.51 ± 4.51	17.66 ± 4.52	18.19 ± 6.58	8.55 ± 3.04	78.72 ± 19.46
Pharmacy Services (n = 73)	12.23 ± 3.17	22.22 ± 4.74	17.73 ± 4.53	18.79 ± 5.86	9.16 ± 3.02	80.14 ± 18.21
*F*	1.25	0.12	0.09	0.82	2.23	0.34
*p*	0.294	0.948	0.965	0.482	0.085	0.795
η^2^	0.012	0.001	0.001	0.021	0.008	0.003
No (n = 214)	12.18 ± 3.47	22.24 ± 5.04	17.50 ± 4.67	18.16 ± 6.31	8.52 ± 3.22	78.61 ± 19.21
Yes (n = 99)	13.01 ± 2.99	22.44 ± 4.48	17.71 ± 4.23	19.65 ± 5.83	9.18 ± 3.23	81.99 ± 17.69
*t*	−2.05	−0.35	−0.37	−1.98	−1.69	−1.49
*p*	0.041	0.728	0.714	0.049	0.092	0.139
Cohen’s d	0.25	0.04	0.05	0.20	0.24	0.18

*t* = Independent samples *t*-test (df = 311); F = One-way analysis of variance (ANOVA) (df = 3, 309). Statistical significance was set at *p* < 0.05.

## Data Availability

The original contributions presented in the study are included in the article, further inquiries can be directed to the corresponding author.
